# Size of an interspecific competitor may be a source of information in reproductive decisions

**DOI:** 10.1093/beheco/arac094

**Published:** 2022-12-13

**Authors:** Reetta Hämäläinen, Panu Välimäki, Jukka T Forsman

**Affiliations:** University of Oulu, Pentti Kaiteran katu 1, 90570 Oulu, Finland; University of Oulu, Pentti Kaiteran katu 1, 90570 Oulu, Finland; University of Oulu, Pentti Kaiteran katu 1, 90570 Oulu, Finland; Natural Resources Institute Finland, Paavo Havaksen tie 3, 90570 Oulu, Finland

**Keywords:** cost of competition, extended phenotype, inter-specific information use, physical phenotype, territory choice

## Abstract

Animals use inter-specific cues as a source of information in decisions-making, but the full costs and benefits of inter-specific information use are unknown. We tested whether pied flycatchers use the body size and clutch size of great tits as cues in their reproductive decisions and what are the possible fitness consequences as a function of great tit size. The size of great tit females associated positively with flycatcher’s probability to settle near a tit nest over a territory further away. Flycatcher egg mass was positively correlated with great tit female size regardless of flycatcher territory choice. However, in flycatchers that had chosen to nest near great tits, the size of nestlings decreased in relation to increasing great tit female size. Our results demonstrate the use of size of inter-specifics as a cue in reproductive decisions and the trade-off between the value of information and costs of competition information users face when using inter-specific information in decision-making.

## INTRODUCTION

The choice of territory is among the most significant decisions animals make and the quality of the territory usually has a considerable effect on individual’s reproductive success (Robinson et al. 1995; Roberts and Norment 1999; Martin and Martin 2001; Rodríquez et al. 2006; Thomson et al. 2006a, 2006b). There is a lot of variation in the quality of territories and to make optimal decisions animals need information about the factors affecting reproductive output, such as amount and quality of food, predation risk and intensity of competition. Animals may acquire the information directly by personally sampling the environment or they may use social information acquired from con- or hetero-specific cues (Danchin et al. 2004; Seppänen et al. 2007; Farine et al. 2015). Often hetero-specific cues are more abundant and obtainable than conspecific cues because often the majority of coexisting individuals are hetero-specifics (Seppänen et al. 2007). Social information use is assumed to be beneficial because it is faster and less costly to gather than obtaining information personally (Dall et al. 2005).

The theory of inter-specific information use (Seppänen et al. 2007) predicts that colonizing individuals are attracted to the presence of competing species if the value of the information they provide exceeds the costs of coexistence. Therefore, species with overlapping resource needs (i.e., putative competitors) may also produce benefits in reproductive decisions if they can be used as a source of information on shared resources. For example, resident species that are ahead in the reproductive cycle compared to migrants can offer them a general indication on habitat quality (Mönkkönen et al. 1990; Forsman et al. 1998). The theory of inter-specific information use predicts that the value of inter-specific social information is a function of the spatial, temporal, and ecological distance between the source and user of information and that the optimal distance depends on the fitness value of information and the cost of competition (Seppänen et al. 2007). However, whether animals evaluate this predicted trade-off in their decision-making and its fitness consequences are unknown.

Recent studies have shown that bird’s nests and eggs are important sources of information in an inter-specific context (Forsman et al. 2002, 2008, 2012; Parejo et al. 2005; Seppänen et al. 2007; Loukola et al. 2012; Kivelä et al. 2014). Migratory birds have been shown to use the nests of resident species as a signal for a good breeding site and they gain fitness benefits by settling close to even their potential competitors (Forsman et al. 2002). Migrant pied flycatchers (*Ficedula hypoleuca*) use the visible clutch size of resident great tits (*Parus major*) as information on how much to invest in their own offspring (Forsman et al. 2008, 2012) and in experiments where flycatchers are enforced to choose between two nest boxes with novel nest-site features, they have been shown to use the clutch size of the demonstrator great tits as a cue for their own decision (Seppänen et al. 2011; Loukola et al. 2012).

The physical phenotype of an individual, such as body size, may also potentially be an important cue in inter-specific information use because it often reflects resource use and competitive ability within species (Maynard Smith and Parker 1976; Johnsson et al. 1999; Candolin and Voigt 2001). Larger individuals are generally more dominant in interference competition (Fretwell and Lucas 1969; Persson 1985) than smaller ones and, hence, according to the ideal despotic distribution model (Fretwell 1972; Parker and Sutherland 1982), occupy high-quality breeding habitats (Maynard Smith and Parker 1976; Johnsson et al. 1999). Indeed, Candolin and Voigt (2001) found that larger three spined stickleback (*Gasterosteus aculeatus*) males defend higher quality habitats, while Brandner et al. (2013) showed that body size correlated positively with competitive ability in an invasive round goby (*Neogobius melanostomus*). Furthermore, compared to clutch size, body size can be a safer and easier cue to observe, because it can be detected from a distance and it does not require approaching and entering the nest, which may result in aggression by the great tit and death in the worst case (Merilä and Wiggins 1995; Forsman et al. 2018). Because both physical phenotype and reproductive effort, such as clutch size, convey information that correlates positively with food resources and habitat quality (Perrins 1965; Dhondt et al. 1992), they can provide a reliable way to estimate the quality of the territory as well as the amount of competition and help to adjust the reproductive decisions accordingly.

Migratory pied flycatchers and resident great tits have similar resource needs, and they are potential competitors (Slagsvold 1975; Minot 1981; Lundberg and Alatalo 1992; Samplonius and Both 2019). Yet, comprehensive correlative and experimental evidence shows that flycatchers (pied flycatcher and collared flycatcher [*Ficedula albicollis*]) use the territory and reproductive investment (number of eggs in the nest) decisions of resident great tits in their own corresponding decisions (e.g., Forsman et al. 2002, 2008, 2012; Kivelä et al. 2014; Samplonius and Both 2019; Morinay et al. 2020). However, clutch size information may not always be a reliable cue for flycatchers. First, great tits have natural variation in egg covering propensity from fully covered to completely visible eggs. Second, Loukola et al. (2014) showed experimentally that great tits actively try to disguise clutch size information from flycatchers by covering their eggs with nest material when flycatcher presence was experimentally simulated, plausibly because flycatcher proximity may result in costs for great tits (Forsman et al. 2007). Thus, compared to the hypothesized body size as a source of information, clutch size includes uncertainty and the relative importance of great tit’s clutch size and body size in flycatcher’s settlement and reproductive investments decisions and the fitness consequences of the decisions are not known.

Here, we test with a field experiment whether migratory pied flycatchers use the body size and nest location (distance to the great tit nest) and/or number of eggs of resident great tits as a source of information in their 1) territory and 2) offspring investment (pied flycatcher clutch size, egg mass) decisions, and 3) whether these decisions result in fitness benefits or costs (pied flycatcher brood size, nestling tarsus length) as a function of great tit body size. We hypothesize that the clutch size and body size of great tits convey both the quality of the breeding habitat and the level of competition within the site. We predict that increasing visible clutch size and body size of great tits positively affect the probability that pied flycatchers prefer to nest in the vicinity of a great tit nest and flycatcher’s reproductive investment. The impact on the flycatcher’s fitness measures (nestling number and size) is predicted to depend on the trade-off between the benefits acquired via habitat quality of the territory and costs of competition with resident great tits and their physical phenotype.

## MATERIALS AND METHODS

### Experimental design

The fieldwork took place in Finland within mixed- and coniferous forests surrounding the city of Oulu (65°01ʹN, 25°28ʹE). Forests in the study area are primarily dominated by Scots pine (*Pinus sylvestris),* spruce (*Picea abies)*, and to some degree by birches (*Betula* spp). The field study was carried out in 2017 and 2018 from May to July. The resident great tit and migratory pied flycatcher have similar breeding site and food requirements especially during the nestling phase (Minot 1981; Lundberg and Alatalo 1992).

**Figure 1:**
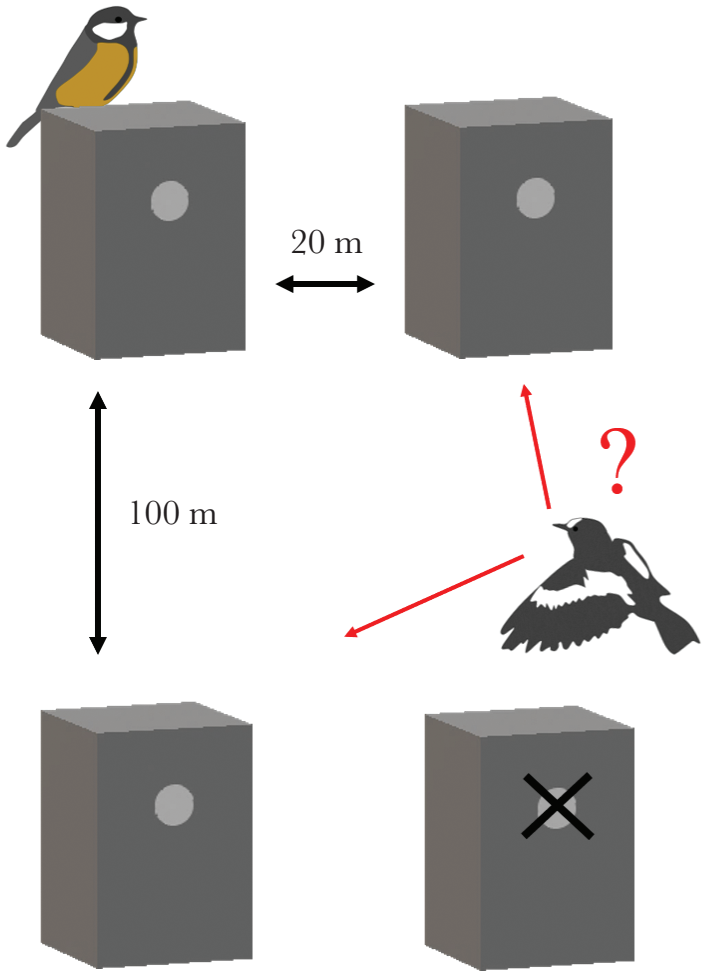
Illustration of the research set-up. After great tits had settled to their nest boxes, two nest boxes were put up for the pied flycatchers to choose from. One near (20 m) and one far (100 m) from the great tit nest box. The similarity of the two location was ensured with a blocked nest box in the further site. These units were replicated so that they were at least 500 m apart in the study area.

In the spring, great tits settled freely in the study area to the available nest boxes situated 500 m apart from one another. Altogether there were 106 available sites. Experimental set-ups were erected when the first pied flycatchers arrived. The experimental design of one sampling unit (set-up) was such that there were two nest boxes available for arriving flycatchers: one box close (20 m) and another further away (100 m) from an active tit nest. We placed an obstructed nest box next to the further (100 m from a tit nest) available nest box to ensure a visual similarity with the nest box that is near the active great tit nest (Figure 1). The box was blocked to prevent a settlement of an additional flycatcher pair. A single set-up was situated in a homogenous forest area and the nest boxes that the pied flycatchers chose from were attached to trees that were of the same species and similar in diameter and height. Thus, there were two unoccupied nest boxes available within the same habitat type, the only difference being the proximity to the occupied great tit nest. Set-ups were replicated so that they were at least 500 m apart to ensure independence of observations. It is possible that there were some great tits breeding in unknown natural cavities near the set-ups, which may have influenced pied flycatcher decisions. However, all set-ups were within managed forests where natural cavities are rare, and none were observed during the field seasons.

Each experimental set-up was monitored at least every second day. This enabled recording the exact time and place (near or further away from great tit) of flycatcher’s territory choice and the visible (number of visible eggs in the nest without moving the possible coverings) and actual clutch size of great tits at the time of the choice. In our study area great tits usually start their nesting ca. 14 days before pied flycatchers and they are already incubating by the time flycatchers start nesting (Loukola et al. 2014). The territory choice of a flycatcher was determined by the appearance of the nest material in the nest boxes. For each nest box, dates of the onset of egg laying, incubation, and hatching of the young were determined. Flycatcher’s breeding investment was determined by measuring the clutch size and the mass of the individual eggs (± 0.1 g digital balance). Mass of the eggs was measured on the day the sixth egg was laid. After 2 days the nest was checked again, and possible additional eggs were measured. Pied flycatcher females were captured from the nest and measured during incubation. When the nestlings had reached the age of 5 days, flycatcher males were captured. Adult birds were captured from the nest box with a flap trap that is placed inside the nest box around the entrance of the box. When the bird enters the nest box the trap closes, and the bird is captured from the nest box. For both sexes, measurements of wing, tail (± 1 mm; with a ruler), and tarsus length (± 0.05 mm; with a caliper) and body mass (± 0.5 g; with Pesola 30 g, string-weighing device) were taken, and the age determined [1 ≤ 2 years (young), 2 = ≥2 years (adult)]. The morphological measurements of flycatcher adults were taken because they are expected to affect offspring investment (clutch size, egg mass), brood and nestling size (Alatalo and Lundberg 1986) and female age was determined because it is a significant factor explaining flycatcher’s territory choice (Loukola et al 2012; Forsman et al. 2022). The same physical measurements were recorded from each nestling at the age of 13 days. Adult great tits were also captured from the nest box after the nestlings reached the age of 5 days and tarsus length of adult great tits was measured in the similar way to provide an estimate of their body size. In the analyses we used tarsus length as a measurement of the physical phenotype because it is more constant than for example body mass, which fluctuates in course of the day in relation to activity, feeding and excreting.

### Analyses

All the analyses were conducted using program R version 3.5.1 (R core team 2018) and in all analyses we used the biologically most meaningful models to test our predictions. We used observed variables, such as initiation of egg laying date and clutch size, as covariates in some analyses to control their effect on other response variables. The number of caught and measured great tit females (*n* = 68) was higher than males (*n* = 63) and to maximize the use of data, two separate models were constructed for all the dependent variables, one model with great tit female and another one with great tit male tarsus length as an explanatory variable.

Generalized linear models [function glm] with logit link function were fitted to determine the variables of great tit body size and clutch size that explain the territory choice of pied flycatchers in relation to great tit nest-site (binary variable: 0 = far, 1 = near). The explanatory variables included in the model testing our predictions were the actual clutch size of great tit and the proportion of visible eggs, great tit tarsus length, pied flycatcher female tarsus length, age of the female pied flycatchers that settled within the specific set-up (Loukola et al. 2012; Jaakkonen et al. 2015; Morinay et al. 2018) and replicate years (2017 and 2018). Pied flycatcher females instead of males were considered as an explanatory variable because females build the nest (Lundberg and Alatalo 1992) and hence have strong impact on the final territory choice (Alatalo et al. 1986). The actual clutch size of great tit, proportion of visible eggs and great tit female/male tarsus length were retained in each model of all the analyses because of their ecological relevance to the research questions and the territory choice of flycatcher was retained in the analyzes determining the variables that explain the offspring investment (clutch size, egg mass) and the fitness benefits and costs (brood size, nestling tarsus length).

Generalized linear models with Poisson link function were fitted to determine the variables that explain the pied flycatcher clutch size (count variable). Explanatory variables included in the model were the interaction of flycatcher’s territory choice (binary variable: 0 = far, 1 = near) and great tit tarsus length (male and female in separate models, see above), the actual clutch size of resident great tit at the moment of flycatcher choice, proportion of visible eggs, female, and male tarsus length of the pied flycatchers that settled within the specific set-up, onset of flycatcher egg laying and replicate years.

Linear mixed models [package nlme (Pinheiro et al. 2019), function lme (R core team 2018)] were fitted to determine the variables that explain the pied flycatcher’s egg mass. Explanatory variables included in the model were otherwise equal to the full model for clutch size (see above) except that the pied flycatcher’s clutch size was included as an explanatory variable and nest was set as a random factor because each egg represents repeated non-independent measures of a particular nesting event.

Generalized linear models with Poisson link function were fitted to determine the variables that explain the number of pied flycatcher nestlings (count variable). Explanatory variables included in the models were the interaction of flycatcher territory choice and great tit female/male tarsus length, tarsus length of female and male pied flycatchers that settled within the specific set-up and replicate years.

Linear mixed models [package nlme (Pinheiro et al. 2019), function lme (R core team 2018)] were fitted to determine the variables that explain flycatcher nestling tarsus length. Explanatory variables included in the model were the same than in the full model for number of nestlings (see above) except that brood size was included as an explanatory variable in analysis of nestling tarsus length because it potentially affects average brood condition and nest was set as a random factor. In all models the statistical significance was determined at 95% confidence interval level.

## RESULTS

**Figure 2:**
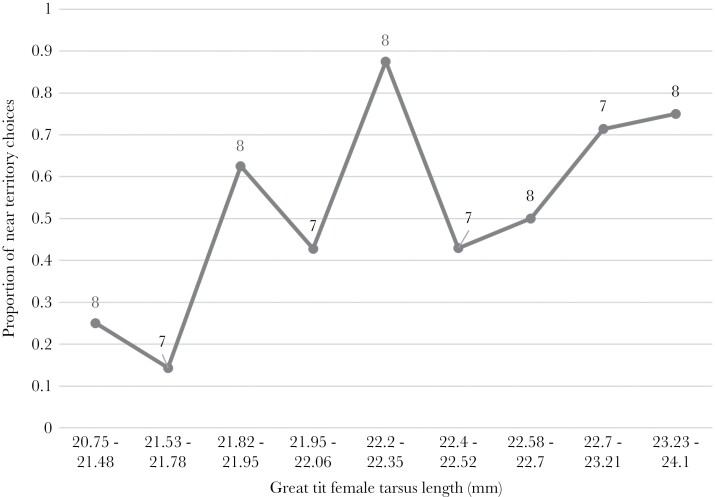
Proportion of pied flycatcher’s choices to nest near a great tit nest in relation to great tit female tarsus length. The scale of great tit female tarsus length is set thus that the great tit females are distributed evenly to each category. In the figure the sample size is marked above each category.

Overall, 79 pied flycatcher territory choices were observed over the two nesting seasons (2017, 2018). The tarsus length of great tit females was associated with pied flycatcher’s territory choice, while the actual clutch size and the proportion of visible eggs had no significant effect. The probability of pied flycatchers settling near a great tit increased when there was a larger great tit female present at the site (Figure 2 and Table 1[a]), while the phenotype of a male great tit did not affect the flycatcher territory choice (Table 1[b]).

**Table 1:** Parameter estimates of the generalized linear models for pied flycatcher (*Ficedula hypoleuca* [FH]) territory choice in relation to phenotype of great tit (*Parus major* [PM]) (a) females [f] and (b) males [m]

Fixed estimates	Estimate	95 % CI	SE	*Z*	*P*-value
(a)					
(Intercept)	−16.902	−45.037 to 9.346	13.674	−1.239	0.217
PM actual clutch size	0.085	−0.313 to 0.506	0.206	0.413	0.680
PM proportion of visible eggs	−1.692	−5.055 to 1.400	1.614	−1.048	0.295
PM f tarsus length	1.072	0.182–2.121	0.489	2.201	0.028
FH_f_tarsus length	−0.310	−1.398 to 0.744	0.538	−0.578	0.564
FH f age (adult)	−0.642	−1.880 to 0.531	0.609	−1.056	0.291
Year (2018)	0.246	−1.719 to 2.316	0.997	0.247	0.805
(b)					
(Intercept)	1.475	−25.654 to 28.882	13.634	0.108	0.914
PM actual clutch size	0.060	−0.340 to 0.478	0.205	0.294	0.769
PM proportion of visible eggs	−1.240	−4.560 to 1.838	1.602	−0.774	0.439
PM m tarsus length	0.160	−0.749 to 1.084	0.458	0.350	0.726
FH_f_tarsus length	−0.217	−1.239 to 0.780	0.507	−0.423	0.668
FH f age (adult)	−0.622	−1.868 to 0.565	0.614	−1.012	0.311
Year (2018)	0.018	−1.916 to 1.975	0.961	0.019	0.985

**Figure 3:**
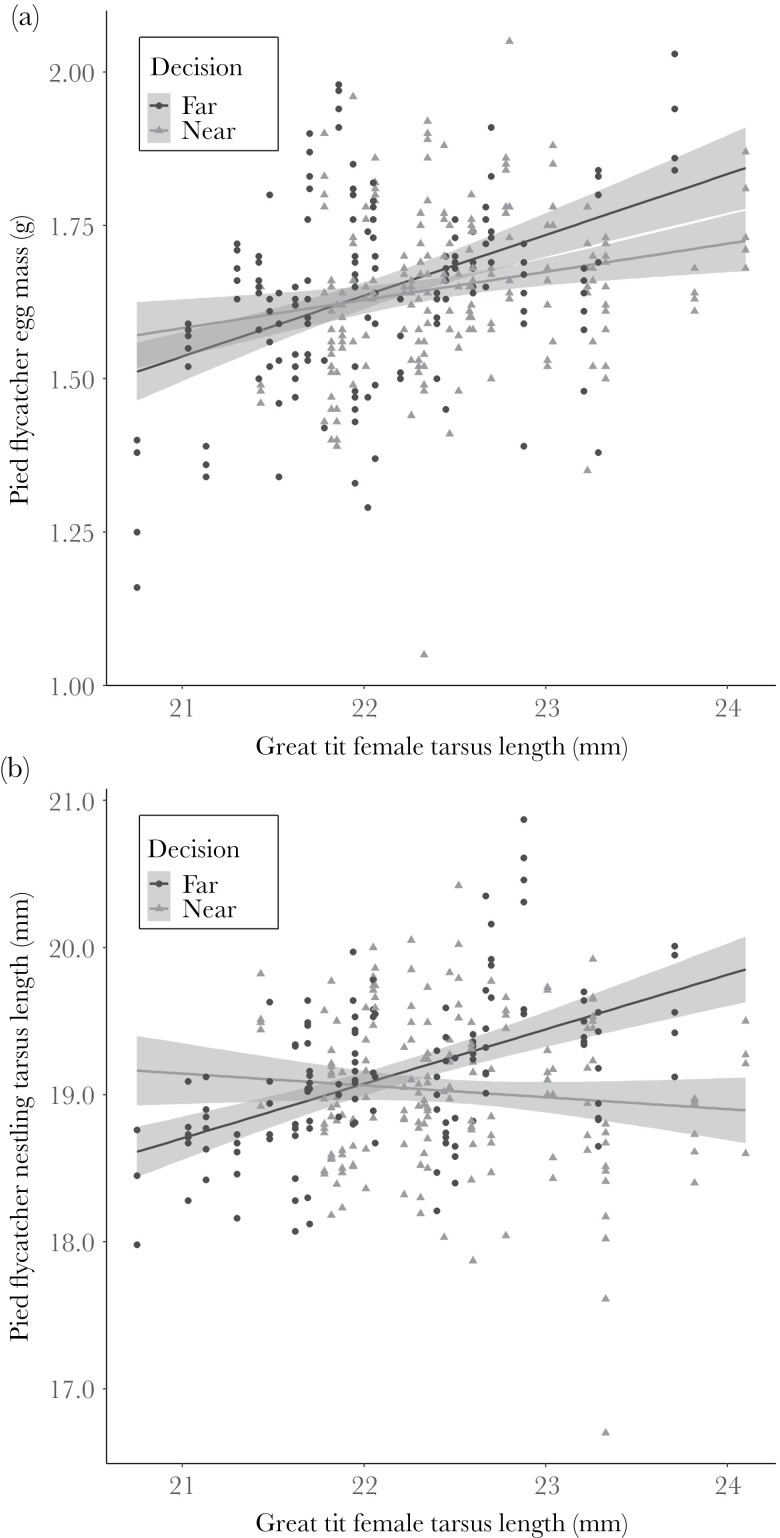
(a) Association between great tit female tarsus length (mm) and pied flycatcher egg mass (g) and (b) between great tit female tarsus length (mm) and pied flycatcher nestling tarsus length (mm) near (gray dashed line) and far (black solid line) from great tit nest with 95% confidence intervals.

The measured variables of the great tit did not explain the pied flycatcher clutch size (Supplementary Table S1a, b). The great tit female tarsus length was associated positively with the average egg mass of flycatchers regardless of flycatcher’s territory choice (far/near great tit nest), while male tarsus length had no effect. The pied flycatcher female tarsus length was associated negatively with the average egg mass (Table 2[a, b] and Figure 3a). The measured variables of great tit did not explain the number of pied flycatcher nestlings (Supplementary Table S2a, b). Tarsus length of the flycatcher nestlings was best explained by tarsus length of both flycatcher parents and interaction between the pied flycatcher territory choice and the great tit female tarsus length (Supplementary Figure S1 and Table 3[a, b]; Figure 3b). Interaction emerged because, among flycatchers that settled near great tits, the tarsus length of nestlings was associated negatively with the tarsus length of the female great tit, while in flycatchers breeding further away from a great tit nest, the association was positive.

**Table 2:** Parameter estimates of linear mixed model for pied flycatcher [FH] egg mass in relation to phenotype of great tit [PM] (a) females [f] and (b) males [m]

Fixed effects	Estimate	95 % CI	SE	DF	*t*-value	*P*-value
(a)						
(Intercept)	0.350	−1.710 to 2.410	1.046	245	0.335	0.738
FH territory choice (near)	0.464	−1.815 to 2.742	1.126	38	0.412	0.683
PM f tarsus length	0.086	0.015–0.157	0.035	38	2.458	0.019
PM actual clutch size	−0.002	−0.014 to 0.011	0.006	38	−0.254	0.801
Proportion of visible eggs	0.014	−0.031 to 0.060	0.022	38	0.636	0.529
FH clutch size	−0.011	−0.055 to 0.033	0.022	38	−0.500	0.620
FH f tarsus length	−0.071	−0.140 to −0.003	0.034	38	−2.119	0.041
FH m tarsus length	0.033	−0.043 to 0.109	0.038	38	0.879	0.385
Egg laying date	0.008	−0.001 to 0.017	0.004	38	1.749	0.088
Year (2018)	−0.034	−0.174 to 0.105	0.069	38	−0.501	0.619
Decision: PM f tarsus length	−0.023	−0.125 to 0.079	0.050	38	−0.449	0.656
Random effects	SD	95 % CI	Residual			
FH nest	0.101	0.079–0.094	0.086			
Fixed effects	Estimate	95 % CI	SE	DF	*t*-value	*P*-value
(b)						
(Intercept)	2.796	−0.042 to 5.634	1.440	223	1.942	0.053
FH territory choice (near)	−0.915	−3.900 to 2.069	1.467	33	−0.624	0.537
PM m tarsus length	−0.071	−0.159 to 0.018	0.043	33	−1.625	0.114
PM actual clutch size	0.001	−0.013 to 0.014	0.007	33	0.081	0.936
Proportion of visible eggs	−0.003	−0.050 to 0.045	0.023	33	−0.109	0.915
FH clutch size	−0.035	−0.089 to 0.018	0.026	33	−1.340	0.190
FH f tarsus length	−0.036	−0.107 to 0.035	0.035	33	−1.022	0.314
FH m tarsus length	0.064	−0.024 to 0.151	0.043	33	1.486	0.147
Egg laying date	0.006	−0.004 to 0.016	0.005	33	1.200	0.239
Year (2018)	−0.028	−0.188 to 0.131	0.078	33	−0.363	0.719
Decision: PM m tarsus length	0.039	−0.091 to 0.170	0.064	33	0.613	0.544
Random effects	SD	95 % CI	Residual			
FH nest	0.109	0.083–0.143	0.089			

**Table 3:** Parameter estimates of linear mixed model for pied flycatcher nestling tarsus length in relation to phenotype of great tit [PM] (a) females [f] and (b) males [m]

Fixed effects	Estimate	95 % CI	SE	DF	*t* value	*P*-value
(a)						
(Intercept)	8.788	1.564–16.011	3.665	216	2.398	0.017
FH territory choice (near)	2.674	−5.186 to 10.534	3.889	40	0.688	0.496
PM m tarsus length	−0.050	−0.282 to 0.182	0.115	40	−0.437	0.665
FH brood size	0.026	−0.053 to 0.104	0.039	40	0.657	0.515
FH f tarsus length	0.268	0.084–0.453	0.091	40	2.946	0.005
FH m tarsus length	0.320	0.118–0.521	0.100	40	3.201	0.003
Year (2018)	−0.021	−0.260 to 0.218	0.118	40	−0.179	0.859
FH territory choice (near): PM m tarsus length	−0.117	−0.460 to 0.226	0.170	40	−0.690	0.494
Random effects	SD	95 % CI	Residual			
FH nest	0.280	0.207–0.379	0.363			

Fixed effects	Estimate	95 % CI	SE	DF	*t* value	*P*-value
(b)						
(Intercept)	5.543	0.523–10.564	2.548	233	2.176	0.031
FH territory choice (near)	5.995	0.250–11.740	2.852	45	2.102	0.041
PM f tarsus length	0.231	0.049–0.413	0.090	45	2.558	0.014
FH brood size	−0.007	−0.076 to 0.062	0.034	45	−0.214	0.832
FH f tarsus length	0.216	0.051–0.381	0.082	45	2.634	0.012
FH m tarsus length	0.227	0.045–0.408	0.090	45	2.520	0.015
Year (2018)	−0.054	−0.253 to 0.145	0.100	45	−0.549	0.586
FH territory choice (near): PM f tarsus length	−0.272	−0.530 to −0.014	0.128	45	−2.126	0.039
Random effects	SD	95 % CI	Residual			
FH nest	0.248	0.183–0.337	0.364			

## DISCUSSION

We tested with a field experiment whether migratory pied flycatchers use the body size and nest location (distance to the nest) of resident great tit and the number of eggs in the nest as a source of information in 1) territory choice and 2) offspring investment decisions, and 3) whether the preferred choice results in fitness benefits or costs as a function of great tit phenotypic size. Our results show that the body size of the great tit female positively affected flycatcher’s probability to settle near the tit nest over the neighboring vacant territory 100 m further away, while the great tit’s nest location or clutch size (number of eggs and proportion of visible eggs) did not explain flycatcher’s reproductive decisions. Flycatcher offspring investment in terms of egg mass was also positively associated with great tit female tarsus length regardless of flycatcher territory choice. However, the fitness effects of the territory choice decision in terms of body size of nestlings (tarsus length) depended on the tarsus length of female great tits. Flycatcher nestlings that grew in nest boxes near large great tit females were smaller than the ones that grew in the further nest boxes.

Our results suggest that pied flycatchers use the body size of a putative competing species to assess the quality of available territories and in the consequent territory choice and offspring investment decisions. Given the positive association between individual’s physical size and quality of territory or habitat (Perrins 1965; Maynard Smith and Parker 1976; Dhondt et al. 1992; Johnsson et al.1999; Candolin and Voigt 2001), the body size of an individual is a reliable and easily observable cue to estimate habitat quality for own decision-making. Compared to the great tit clutch size information, which our previous studies have shown flycatchers to use in offspring investment and nest-site characteristics choice decisions (Seppänen et al. 2011; Forsman et al. 2012; Loukola et al. 2013), using body size as a cue is less risky because it excludes the mortal risk of entering a tit nest box due to great tit aggression (Merilä and Wiggins 1995; Forsman et al. 2018).

Because we did not manipulate the location of great tit nests, we cannot fully exclude the possibility that flycatchers made the settlement and reproductive investment decisions independently based on their own assessment on territory quality or depending on the phenotype (tarsus length) of the great tit relative to their own phenotype resulting in non-random settlement. However, we argue that the most likely explanation is that flycatchers actively used great tit body size as a cue in reproductive decisions. First, it is difficult to find more likely explanation than active selection for the result that flycatchers preferred the territory near the larger great tits with better competitive abilities when there was a vacant territory just 100 m further away. Second the tarsus length of the pied flycatcher female and male was taken into consideration in the model fitted for territory choice and it did not have an effect, suggesting that the preference for breeding close to large great tits does not vary as a function of flycatcher phenotype (tarsus length). Third, the location of experimental set-ups was systematically determined (set-ups were approximately 500 m apart) and nest boxes were placed in habitats preferred by pied flycatchers suggesting that habitat quality variation among set-ups is not large. The evidence on the effect of habitat quality on reproductive decisions is mixed. Some studies have shown that it reflects to clutch size or offspring numbers (Perrins 1965; Dhondt et al. 1992; Siikamäki 1995; Risch et al. 2007), while others have reported less conclusive results (Martin 1987; Svensson and Nilsson 1995). Birds are known to adjust offspring investment relative to nest predation, which is the major mortality factor among birds (Martin 1995). For example, Zanette et al. (2011) found that the perception of predation risk reduced the number of offspring produced per year by 40% and Fontaine and Martin (2006) experimentally showed in eight passerine species that birds can increase egg mass up to 20% in predator removal treatment compared to control while no difference was observed in clutch size. In our study, the difference in pied flycatcher egg mass according to the estimated regression line between the far ends of great tit female tarsus length (0.23 g) is 13.9 % of the mean egg weight (1.65 g), when the egg mass data of far and near nest boxes is combined (Figure 3). In this context, the observed difference in flycatcher egg mass as a function of the body mass of great tit female is remarkable and most likely not resulting from habitat quality differences and suggest that flycatchers have adjusted their offspring investment relative to the body size of neighboring great tit. We thus conclude that the body size of other species with overlapping resource needs can be used as a source of information in reproductive decisions.

Great tit male body size did not explain the flycatcher settlement and offspring investment decisions. One potential explanation is that because females spend more time on the breeding sites (egg laying, incubating) they are more likely to be observed than males and therefore their size is more probable to explain the flycatcher decision. Female birds also invest more in breeding (Møller and Birkhead 1993; Schwagmeyer et al. 1999) and therefore cues they offer could potentially be more reliable.

Flycatchers preferred to breed close to large great tits but its implications for the condition of flycatcher nestlings varied between near and further boxes. The association between great tit female tarsus length and flycatcher nestling’s tarsus length growing far from the great tit nests was positive. In contrast, in the nest boxes near a great tit, the nestlings had a shorter tarsus length than nestlings in further nest boxes with similar sized females if the great tit female had a tarsus length of 22mm or longer (Figure 3b). Apparently growing in the vicinity of a large putative competitor has increased costs of competition that, despite similar investment in egg mass, resulted in nestlings that were in poorer physical condition (in terms of tarsus length) compared to the offspring whose parents chose to breed further away from the great tit nest. According to the estimated regression lines, the difference in tarsus lengths between different territory choices (near/far) in sites with bigger great tit female is 0.81 mm, which is 4.23% of the mean nestling tarsus length (19.14 mm) (Figure 3b). The difference is biologically significant and smaller size may negatively affect survival and future breeding success (Garnett 1981; Sanz and García-Navas 2011).

Our results suggest that flycatchers preference to nest near larger great tit females were not optimal. The explanation for this remains unknown but plausible reasons are annual variation in weather conditions that plausibly may affect food resources and productivity of environment. Normally flycatcher’s decision to nest near larger great tit females could be beneficial and cause negative fitness consequences only in certain years. Off the study years, spring and early summer in 2017 was abnormally cold (snowing in May and June). We examined the effect of this annual variation by analyzing the data separately between the study seasons (Supplementary Table S3–S12). In 2017 the tarsus lengths of the nestlings were positively associated with great tit female tarsus length regardless of the territory choice (Supplementary Table S11 and Figure S2a), while in 2018 there was a negative association between pied flycatcher nestling tarsus length and great tit female tarsus length in the nests near a great tit nest (Supplementary Table S12 and Figure S2b). Abnormally poor breeding conditions thus seem not to explain the quantitatively different patterns but there clearly is annual variation in outcomes in breeding close vs. further away from a putative competitor. Our previous studies have shown that flycatchers prefer to breed close to tit nests that result in fitness benefits in terms of larger nestlings (Forsman et al. 2002, 2007). These studies were conducted in more productive environments (lush riparian deciduous forest) than the present study (mixed or coniferous forest), which may explain the slightly different results. In concert, the current and our previous results (Forsman et al. 2002, 2007) suggest that profitability of the preference to breed near large competitors varies among years and plausibly depends on the biotic conditions during a reproductive period. We suggest that the fitness consequences of decision-making based on inter-specific information use vary and depend upon the prevailing environmental conditions, resulting in inconsistent selection pressures between different years. Such an environmental dependent selection pressure is indeed common, for example in the context of sexual selection (Higginson and Reader 2009). In the collared flycatchers, relative fitness consequences as a function of male ornamentation were found to depend on the moisture conditions of the breeding period (Robinson et al. 2012). Indeed, other studies have found plasticity in mate choice across breading season with different species (Lynch et al. 2005; Chaine and Lyon 2008). Lark buntings (*Calamospiza melanocorys*) sexual selection on male traits varied across years and the female preference changed according to how the male trait predicted female reproductive success each year (Chaine and Lyon 2008).

Our results and previous studies with similar experimental set-up (Forsman et al. 2002, 2007) demonstrate that both physical and extended phenotype (nest and eggs) of great tit could act as a cue of territory quality and that animals can use these inter-specific cues in their decision-making. Birds nest (Schaedelin and Taborsky 2009; Jelinek et al. 2016) and eggs (Cassey et al. 2011; Nagy et al. 2019) are a traditional example of extended phenotypes (Dawkins 1982), and it is known that extended phenotypes are widely used in signaling (e.g., mate selection, prey attraction and protection) (Hansell 1984; Herberstein et al. 2000; Quader 2005). The significance of extended phenotypes as signals has been mostly studied in the context of conspecifics (Schaedelin and Taborsky 2009), but our results show that extended phenotypes can also be used as a source of information also in inter-specific context. We put forward a hypothesis that if extended phenotypes are associated with resource quality or distribution, they may be used as a source of information by others (con- and hetero-specifics) and they can have significant consequences for spatial distribution of species and community structure.

Our results on the preference to breed and enhanced investment in offspring in the vicinity of a physically large putative competitor does not match with rather static predictions of traditional theories of coexistence and competition (e.g., Fretwell and Lucas 1969; MacArthur 1972; Persson 1985; Gustafsson 1987; Balance et al. 1997; Martin and Martin 2001; Rolo and Moreno 2012), which expect avoidance of potential competitors. Rather, our results are in line with the predictions of inter-specific information use (Seppänen et al. 2007) whereby the optimal choices depend on the distance in time, space and ecology between the information user and information source and the potential value of information may trade-off with costs of negative interactions, such as competition. We have demonstrated here for the first time the usage the physical phenotype of other species as a source of information in reproductive decisions, and the trade-off situation between the value of information (distance to the information source; Seppänen et al. 2007) and costs of competition information users face in decision-making. These findings highlight the importance of trade-off between the value of inter-specific information and costs of competition in territory and reproductive decisions, ultimately affecting the distribution of individuals in space and their interactions.

## Supplementary Material

arac094_suppl_Supplementary_Material

## Notes

We thank Mahdi Aminikhah, Tuomas Kankaanpää, Mira Kajanus, Sami Kivelä, Thomas Merckx, and Mahtab Yazdanian for their help in improving previous versions of the manuscript.
