# Supplementary material for: Size of an interspecific competitor may be a source of information in reproductive decisions
Source: Behav Ecol. 2022 Dec 13;34(1):33–41. doi: 10.1093/beheco/arac094 (PMC9918860; doi:10.1093/beheco/arac094)
Supplement: arac094_suppl_Supplementary_Material [file arac094_suppl_supplementary_material.docx]

**Supplementary Material**

Size of an interspecific competitor may be a source of information in reproductive decisions

^1^Reetta Hämäläinen, ^1^Panu Välimäki, ^1,2^Jukka T. Forsman

^1^University of Oulu, Oulu, Finland. ^2^Natural Resources Institute Finland, Oulu, Finland

**This file includes**

**Tables S1-S12**

**Figure S1-S2**

Table S1. Parameter estimates of the generalized linear models for pied flycatcher [FH] clutch size (eggs) in relation to phenotype of great tit [PM] (a) females [f] and (b) males [m].

a)

| **Fixed estimates** | **Estimate** | **95 % CI** | **SE** | **Z** | **p-value** |
| --- | --- | --- | --- | --- | --- |
| (Intercept) | -0.895 | -7.930 – 6.126 | 3.585 | -0.250 | 0.803 |
| FH territory choice (near) | 0.888 | -7.184 – 9.007 | 4.129 | 0.215 | 0.830 |
| PM f tarsus length | 0.019 | -0.230 – 0.265 | 0.126 | 0.149 | 0.882 |
| PM actual clutch size | -0.019 | -0.102 – 0.061 | 0.041 | -0.467 | 0.640 |
| PM proportion of visible eggs | 0.142 | -0.463 – 0.756 | 0.311 | 0.458 | 0.647 |
| FH f tarsus length | 0.085 | -0.142 – 0.318 | 0.117 | 0.726 | 0.468 |
| FH m tarsus length | 0.044 | -0.206 – 0.294 | 0.128 | 0.341 | 0.733 |
| FH egg laying date | -0.008 | -0.037 – 0.020 | 0.015 | -0.564 | 0.572 |
| Year (2018) | 0.062 | -0.421 – 0.559 | 0.250 | 0.248 | 0.804 |
| FH territory choice (near):  PM f tarsus length | -0.038 | -0.402 – 0.324 | 0.185 | -0.206 | 0.837 |

b)

| **Fixed estimates** | **Estimate** | **95 % CI** | **SE** | **Z** | **p-value** |
| --- | --- | --- | --- | --- | --- |
| (Intercept) | 1.588 | -8.137 – 11.252 | 4.937 | 0.322 | 0.748 |
| FH territory choice (near) | -1.577 | -11.643 – 8.265 | 5.075 | -0.311 | 0.756 |
| PM m tarsus length | -0.107 | -0.409 – 0.190 | 0.152 | -0.703 | 0.482 |
| PM actual clutch size | -0.019 | -0.111 – 0.071 | 0.047 | -0.418 | 0.676 |
| PM proportion of visible eggs | 0.103 | -0.587 – 0.799 | 0.353 | 0.292 | 0.770 |
| FH m tarsus length | 0.068 | -0.164 – 0.307 | 0.120 | 0.567 | 0.571 |
| FH m tarsus length | 0.075 | -0.178 – 0.329 | 0.129 | 0.579 | 0.562 |
| FH egg laying date | -0.004 | -0.034 – 0.026 | 0.015 | -0.274 | 0.784 |
| Year (2018) | 0.077 | -0.429 – 0.592 | 0.260 | 0.297 | 0.766 |
| FH territory choice (near):  PM m tarsus length | 0.071 | -0.359 – 0.511 | 0.222 | 0.320 | 0.749 |

Table S2. Parameter estimates of the generalized linear mixed models for the number of pied flycatchers [FH] nestlings in relation to phenotype of great tit [PM] (a) females [f] and (b) males [m].

a)

| **Fixed estimates** | **Estimate** | **95 % CI** | **SE** | **Z** | **p-value** |
| --- | --- | --- | --- | --- | --- |
| (Intercept) | -1.121 | -8.206 – 5.943 | 3.609 | -0.311 | 0.756 |
| FH territory choice (near) | 2.622 | -5.378 – 10.664 | 4.090 | 0.641 | 0.521 |
| PM f tarsus length | 0.097 | -0.155 – 0.348 | 0.128 | 0.754 | 0.451 |
| FH f tarsus length | 0.055 | -0.174 – 0.288 | 0.118 | 0.466 | 0.641 |
| FH m tarsus length | -0.022 | -0.276 – 0.232 | 0.130 | -0.167 | 0.868 |
| Year (2018) | 0.101 | -0.177 – 0.382 | 0.143 | 0.709 | 0.478 |
| FH territory choice (near):  PM f tarsus length | -0.119 | -0.479 – 0.240 | 0.183 | -0.649 | 0.516 |

b)

| **Fixed estimates** | **Estimate** | **95 % CI** | **SE** | **Z** | **p-value** |
| --- | --- | --- | --- | --- | --- |
| (Intercept) | 2.072 | -7.430 – 11.617 | 4.852 | 0.427 | 0.669 |
| FH territory choice (near) | -3.564 | -13.678 – 6.338 | 5.103 | -0.698 | 0.485 |
| PM m tarsus length | -0.064 | -0.364 – 0.229 | 0.151 | -0.422 | 0.673 |
| FH f tarsus length | 0.031 | -0.200 – 0.268 | 0.119 | 0.262 | 0.793 |
| FH m tarsus length | 0.026 | -0.226 – 0.278 | 0.129 | 0.200 | 0.841 |
| Year (2018) | 0.034 | -0.269 – 0.344 | 0.156 | 0.219 | 0.826 |
| FH territory choice (near):  PM m tarsus length | 0.155 | -0.277 – 0.596 | 0.223 | 0.695 | 0.487 |

Table S3 Parameter estimates of the generalized linear models for pied flycatcher [FH] territory choice in relation to phenotype of great tit [PM] (a) females [f] and (b) males [m] in year 2017.

a)

| **Fixed estimates** | **Estimate** | **95 % CI** | **SE** | **Z** | **p-value** |
| --- | --- | --- | --- | --- | --- |
| (Intercept) | -19.680 | -64.366 – 18.612 | 20.396 | -0.965 | 0.335 |
| PM actual clutch size | 0.205 | -0.350 – 0.846 | 0.289 | 0.710 | 0.478 |
| PM proportion of visible eggs | -2.286 | -7.284 – 1.960 | 2.240 | -1.020 | 0.308 |
| PM f tarsus length | 0.624 | -0.793 – 2.224 | 0.740 | 0.843 | 0.399 |
| FH f tarsus length | 0.401 | -1.285 – 2.129 | 0.834 | 0.480 | 0.631 |
| FH f age (adult) | -2.225 | -5.711 – 0.115 | 1.389 | -1.601 | 0.109 |

b)

| **Fixed estimates** | **Estimate** | **95 % CI** | **SE** | **Z** | **p-value** |
| --- | --- | --- | --- | --- | --- |
| (Intercept) | 18.002 | -244.601 - NA | 2813.392 | 0.006 | 0.995 |
| PM actual clutch size | 0.108 | -0.581 – 0.828 | 0.340 | 0.316 | 0.752 |
| PM proportion of visible eggs | -1.400 | -6.460 – 3.317 | 2.364 | -0.592 | 0.554 |
| PM m tarsus length | -0.718 | -2.697 – 0.780 | 0.825 | -0.870 | 0.384 |
| FH f tarsus length | 0.872 | -1.098 – 3.152 | 1.037 | 0.841 | 0.401 |
| FH f age (adult) | -18.712 | NA – 192.348 | 2813.310 | -0.007 | 0.995 |

Table S4. Parameter estimates of the generalized linear models for pied flycatcher [FH] territory choice in relation to phenotype of great tit [PM] (a) females [f] and (b) males [m] in year 2018.

a)

| **Fixed estimates** | **Estimate** | **95 % CI** | **SE** | **Z** | **p-value** |
| --- | --- | --- | --- | --- | --- |
| (Intercept) | -2.482 | -174.948 - NA | 2530.278 | -0.001 | 0.9992 |
| PM actual clutch size | -0.179 | -0.905 – 0.500 | 0.349 | -0.511 | 0.6092 |
| PM proportion of visible eggs | -21.357 | NA – 336.377 | 2530.192 | -0.008 | 0.9933 |
| PM f tarsus length | 1.971 | 0.511 – 4.072 | 0.875 | 2.252 | 0.0243 |
| FH f tarsus length | -0.969 | -2.713 – 0.581 | 0.818 | -1.185 | 0.2362 |
| FH f age (adult) | 0.205 | -1.403 – 1.850 | 0.815 | 0.252 | 0.8010 |

b)

| **Fixed estimates** | **Estimate** | **95 % CI** | **SE** | **Z** | **p-value** |
| --- | --- | --- | --- | --- | --- |
| (Intercept) | 4.471 | -220.681 – NA | 3092.591 | 0.001 | 0.999 |
| PM actual clutch size | 0.098 | -0.515 – 0.720 | 0.307 | 0.317 | 0.751 |
| PM proportion of visible eggs | -19.579 | NA – 366.465 | 3092.47 | -0.006 | 0.995 |
| PM m tarsus length | 1.167 | -0.378 – 3.011 | 0.838 | 1.393 | 0.164 |
| FH f tarsus length | -0.666 | -2.328 – 0.872 | 0.797 | -0.836 | 0.403 |
| FH f age (adult) | 0.514 | -1.066 – 2.217 | 0.820 | 0.627 | 0.531 |

Table S5. Parameter estimates of the generalized linear models for pied flycatcher [FH] clutch size (eggs) in relation to phenotype of great tit [PM] (a) females [f] and (b) males [m] in year 2017.

a)

| **Fixed estimates** | **Estimate** | **95 % CI** | **SE** | **Z** | **p-value** |
| --- | --- | --- | --- | --- | --- |
| (Intercept) | 1.345 | -11.271 – 14.330 | 6.510 | 0.207 | 0.836 |
| FH settlement decision | 1.901 | -10.093 – 14.181 | 6.178 | 0.308 | 0.758 |
| PM f tarsus length | 0.022 | -0.351 – 0.380 | 0.186 | 0.117 | 0.907 |
| PM actual clutch size | -0.033 | -0.152 – 0.082 | 0.060 | -0.546 | 0.585 |
| PM proportion of visible eggs | 0.211 | -0.592 – 1.031 | 0.413 | 0.511 | 0.609 |
| FH f tarsus length | 0.056 | -0.283 – 0.409 | 0.176 | 0.317 | 0.751 |
| FH m tarsus length | -0.043 | -0.475 – 0.384 | 0.219 | -0.196 | 0.845 |
| FH egg laying date | -0.009 | -0.060 – 0.048 | 0.027 | -0.322 | 0.748 |
| FH territory choice (near): PM f tarsus length | -0.084 | -0.630 – 0.448 | 0.274 | -0.308 | 0.758 |

b)

| **Fixed estimates** | **Estimate** | **95 % CI** | **SE** | **Z** | **p-value** |
| --- | --- | --- | --- | --- | --- |
| (Intercept) | 2.229 | -12.560 – 16.653 | 7.420 | 0.300 | 0.764 |
| FH settlement decision | 0.442 | -14.069 – 14.958 | 7.389 | 0.060 | 0.952 |
| PM m tarsus length | -0.114 | -0.615 – 0.392 | 0.256 | -0.445 | 0.656 |
| PM actual clutch size | -0.013 | -0.164 – 0.392 | 0.076 | -0.168 | 0.866 |
| PM proportion of visible eggs | 0.035 | -0.983 – 1.067 | 0.521 | 0.067 | 0.947 |
| FH f tarsus length | 0.117 | -0.300 – 0.542 | 0.214 | 0.548 | 0.583 |
| FH m tarsus length | 0.004 | -0.463 – 0.463 | -0.235 | 0.016 | 0.987 |
| FH egg laying date | -0.004 | -0.061 – 0.056 | 0.030 | -0.136 | 0.892 |
| FH territory choice (near): PM m tarsus length | -0.022 | -0.651 – 0.608 | 0.321 | -0.068 | 0.946 |

Table S6. Parameter estimates of the generalized linear models for pied flycatcher [FH] clutch size (eggs) in relation to phenotype of great tit [PM] (a) females [f] and (b) males [m] in year 2018.

a)

| **Fixed estimates** | **Estimate** | **95 % CI** | **SE** | **Z** | **p-value** |
| --- | --- | --- | --- | --- | --- |
| (Intercept) | -1.352 | -10.351 – 7.839 | 4.630 | -0.292 | 0.770 |
| FH settlement decision | -0.282 | -12.401 – 11.955 | 6.204 | -0.045 | 0.964 |
| PM f tarsus length | -0.022 | -0.392 – 0.359 | 0.191 | -0.114 | 0.910 |
| PM actual clutch size | -0.008 | -0.135 – 0.117 | 0.064 | -0.131 | 0.896 |
| PM proportion of visible eggs | 0.145 | -0.921 – 1.238 | 0.549 | 0.264 | 0.792 |
| FH f tarsus length | 0.110 | -0.224 – 0.453 | 0.172 | 0.640 | 0.522 |
| FH m tarsus length | 0.087 | -0.250 – 0.424 | 0.172 | 0.508 | 0.611 |
| FH egg laying date | -0.009 | -0.049 – 0.031 | 0.020 | -0.448 | 0.654 |
| FH territory choice (near): PM f tarsus length | 0.016 | -0.535 – 0.562 | 0.280 | 0.058 | 0.954 |

b)

| **Fixed estimates** | **Estimate** | **95 % CI** | **SE** | **Z** | **p-value** |
| --- | --- | --- | --- | --- | --- |
| (Intercept) | 5.715 | -13.822 – 24.890 | 9.861 | 0.580 | 0.562 |
| FH settlement decision | -6.888 | -23.665 – 9.556 | 8.465 | -0.814 | 0.416 |
| PM m tarsus length | -0.266 | -0.861 – 0.340 | 0.306 | -0.868 | 0.386 |
| PM actual clutch size | -0.015 | -0.162 – 0.134 | 0.075 | -0.205 | 0.838 |
| PM proportion of visible eggs | 0.116 | -1.180 – 1.403 | 0.657 | 0.177 | 0.860 |
| FH f tarsus length | -0.016 | -0.439 – 0.410 | 0.216 | -0.072 | 0.943 |
| FH m tarsus length | 0.125 | -0.218 – 0.471 | 0.175 | 0.710 | 0.478 |
| FH egg laying date | -0.001 | -0.042 – 0.040 | 0.021 | -0.049 | 0.961 |
| FH territory choice (near): PM m tarsus length | 0.307 | -0.417 -1.044 | 0.372 | 0.824 | 0.410 |

Table S7. Parameter estimates of linear mixed model for pied flycatcher [FH] egg mass in relation to phenotype of great tit [PM] (a) females [f] and (b) males [m] in year 2017.

a)

| **Fixed effects** | **Estimate** | **95 % CI** | | **SE** | | **DF** | | **t-value** | | | **p-value** | | |  |
| --- | --- | --- | --- | --- | --- | --- | --- | --- | --- | --- | --- | --- | --- | --- |
| (Intercept) | -0.976 | -4.041 – 2.090 | | 1.546 | | 107 | | -0.631 | | | 0.529 | | |  |
| FH territory choice (near) | -0.077 | -3.356 – 3.201 | | 1.505 | | 12 | | -0.051 | | | 0.960 | | |  |
| PM f tarsus length | 0.053 | -0.045 – 0.152 | | 0.045 | | 12 | | 1.183 | | | 0.260 | | |  |
| PM actual clutch size | -0.021 | -0.053 – 0.012 | | 0.015 | | 12 | | -1.439 | | | 0.176 | | |  |
| Proportion of visible eggs | 0.145 | -0.075 – 0.364 | | 0.101 | | 12 | | 1.435 | | | 0.177 | | |  |
| FH clutch size | 0.007 | -0.059 – 0.072 | | 0.030 | | 12 | | 0.217 | | | 0.832 | | |  |
| FH f tarsus length | -0.039 | -0.130 – 0.053 | | 0.042 | | 12 | | -0.922 | | | 0.375 | | |  |
| FH m tarsus length | 0.084 | -0.033 – 0.200 | | 0.053 | | 12 | | 1.467 | | | 0.143 | | |  |
| FH laying date | 0.018 | 0.003 – 0.033 | | 0.007 | | 12 | | 2.561 | | | 0.025 | | |  |
| FH territory choice (near): PM f tarsus length | 0.001 | -0.145 – 0.147 | 0.067 | | 12 | | 0.015 | | | 0.988 | | |  |  |
| **Random effects** | **SD** | **95 % CI** | **Residual** | |  | | | |  | | |  | | |
| FH nest | 0.091 | 0.057 – 0.144 | 0.088 | |  | | | |  | | |  | | |

b)

| **Fixed effects** | **Estimate** | **95 % CI** | | **SE** | | **DF** | | **t-value** | | | **p-value** | | |  |
| --- | --- | --- | --- | --- | --- | --- | --- | --- | --- | --- | --- | --- | --- | --- |
| (Intercept) | 1.958 | -1.616 – 5.532 | | 1.801 | | 99 | | 1.087 | | | 0.280 | | |  |
| FH territory choice (near) | -3.028 | -6.914 – 0.859 | | 1.744 | | 10 | | -1.736 | | | 0.113 | | |  |
| PM m tarsus length | -0-097 | -0.248 – 0.053 | | 0.067 | | 10 | | -1445 | | | 0.179 | | |  |
| PM actual clutch size | -0.019 | -0.059 – 0.022 | | 0.018 | | 10 | | -1.038 | | | 0.324 | | |  |
| Proportion of visible eggs | 0.146 | -0.126 – 0.419 | | 0.122 | | 10 | | 1.196 | | | 0.259 | | |  |
| FH clutch size | -0.016 | -0.110 – 0.078 | | 0.042 | | 10 | | -0.376 | | | 0.715 | | |  |
| FH f tarsus length | 0.000 | -0.126 – 0.126 | | 0.057 | | 10 | | 0.006 | | | 0.996 | | |  |
| FH m tarsus length | 0.078 | -0.047 – 0.203 | | 0.056 | | 10 | | 1.385 | | | 0.196 | | |  |
| FH laying date | 0.018 | 0.002 – 0.034 | | 0.007 | | 10 | | 2.542 | | | 0.029 | | |  |
| FH territory choice (near): PM f tarsus length | 0.128 | -0.041 – 0.296 | 0.076 | | 10 | | 1.691 | | | 0.122 | | |  |  |
| **Random effects** | **SD** | **95 % CI** | **Residual** | |  | | | |  | | |  | | |
| FH nest | 0.090 | 0.054 -0.150 | 0.090 | |  | | | |  | | |  | | |

Table S8. Parameter estimates of linear mixed model for pied flycatcher [FH] egg mass in relation to phenotype of great tit [PM] (a) females [f] and (b) males [m] in year 2018.

a)

| **Fixed effects** | **Estimate** | **95 % CI** | | **SE** | | **DF** | | **t-value** | | | **p-value** | | |  |
| --- | --- | --- | --- | --- | --- | --- | --- | --- | --- | --- | --- | --- | --- | --- |
| (Intercept) | 0.772 | -2.106 – 3.649 | | 1.455 | | 138 | | 0.530 | | | 0.597 | | |  |
| FH territory choice (near) | 2.309 | -1.731 – 6.348 | | 1.915 | | 17 | | 1.206 | | | 0.244 | | |  |
| PM f tarsus length | 0.121 | -0.007 – 0.250 | | 0.061 | | 17 | | 1.987 | | | 0.063 | | |  |
| PM actual clutch size | 0.006 | -0.023 – 0.034 | | 0.014 | | 17 | | 0.426 | | | 0.678 | | |  |
| Proportion of visible eggs | -0.001 | -0.067 – 0.064 | | 0.031 | | 17 | | -0.040 | | | 0.969 | | |  |
| FH clutch size | -0.036 | -0.111 – 0.040 | | 0.035 | | 17 | | -0.99 | | | 0.334 | | |  |
| FH f tarsus length | -0.095 | -0.218 – 0.029 | | 0.058 | | 17 | | -1.620 | | | 0.124 | | |  |
| FH m tarsus length | 0.006 | -0.113 – 0.125 | | 0.056 | | 17 | | 0.103 | | | 0.919 | | |  |
| FH laying date | 0.001 | -0.013 – 0.015 | | 0.007 | | 17 | | 0.195 | | | 0.848 | | |  |
| FH territory choice (near): PM f tarsus length | -0.105 | -0.287 – 0.077 | 0.086 | | 17 | | -1.222 | | | 0.238 | | |  |  |
| **Random effects** | **SD** | **95 % CI** | **Residual** | |  | | | |  | | |  | | |
| FH nest | 0.111 | 0.076 – 0.160 | 0.085 | |  | | | |  | | |  | | |

b)

| **Fixed effects** | **Estimate** | **95 % CI** | | **SE** | | **DF** | | **t-value** | | | **p-value** | | |  |
| --- | --- | --- | --- | --- | --- | --- | --- | --- | --- | --- | --- | --- | --- | --- |
| (Intercept) | 6.873 | 2.350 – 11.395 | | 2.285 | | 124 | | 3.008 | | | 0.003 | | |  |
| FH territory choice (near) | -2.244 | -7.430 – 2.943 | | 2.418 | | 14 | | -0.928 | | | 0.370 | | |  |
| PM m tarsus length | -0.180 | -0.340 – -0.021 | | 0.074 | | 14 | | -2.430 | | | 0.030 | | |  |
| PM actual clutch size | 0.010 | -0.013 – 0.032 | | 0.011 | | 14 | | 0.926 | | | 0.370 | | |  |
| Proportion of visible eggs | 0.005 | -0.061 – 0.071 | | 0.031 | | 14 | | 0.152 | | | 0.881 | | |  |
| FH clutch size | -0.079 | -0.151 – -0-007 | | 0.034 | | 14 | | -2.350 | | | 0.034 | | |  |
| FH f tarsus length | -0.123 | -0.236 – -0.009 | | 0.053 | | 14 | | -2.318 | | | 0.036 | | |  |
| FH m tarsus length | 0.076 | -0.047 – 0.199 | | 0.058 | | 14 | | 1.320 | | | 0.208 | | |  |
| FH laying date | 0.004 | -0.008 – 0.016 | | 0.006 | | 14 | | 0.708 | | | 0.491 | | |  |
| FH territory choice (near): PM m tarsus length | 0.101 | -0.128 – 0.329 | 0.107 | | 14 | | 0.916 | | | 0.360 | | |  |  |
| **Random effects** | **SD** | **95 % CI** | **Residual** | |  | | | |  | | |  | | |
| FH nest | 0.092 | 0.060 – 0.142 | 0.088 | |  | | | |  | | |  | | |

Table S9. Parameter estimates of the generalized linear mixed models for the number of pied flycatchers [FH] nestlings in relation to phenotype of great tit [PM] (a) females [f] and (b) males [m] in year 2017.

a)

| **Fixed estimates** | **Estimate** | **95 % CI** | **SE** | **Z** | **p-value** |
| --- | --- | --- | --- | --- | --- |
| (Intercept) | 2.948 | -9.896 – 16.226 | 6.646 | 0.444 | 0.657 |
| FH territory choice (near) | 1.445 | -10.613 – 13.811 | 6.212 | 0.233 | 0.816 |
| PM f tarsus length | 0.030 | -0.345 – 0.395 | 0.188 | 0.164 | 0.871 |
| FH territory choice | 0.079 | -0.268 – 0.444 | 0.181 | 0.434 | 0.664 |
| FH m tarsus length | -0.174 | -0.625 – 0.254 | 0.223 | -0.779 | 0.436 |
| FH territory choice (near): PM f tarsus length | -0.072 | -0.621 – 0.464 | 0.276 | -0.261 | 0.794 |

b)

| **Fixed estimates** | **Estimate** | **95 % CI** | **SE** | **Z** | **p-value** |
| --- | --- | --- | --- | --- | --- |
| (Intercept) | 6.680 | -7.215 – 20.453 | 7.041 | 0.949 | 0.343 |
| FH territory choice (near) | -3.991 | -18.080 – 9.971 | 7.139 | -0.559 | 0.576 |
| PM m tarsus length | -0.128 | -0.551 – 0.295 | 0.215 | -0.591 | 0.554 |
| FH territory choice | 0.094 | -0.276 – 0.496 | 0.197 | 0.479 | 0.632 |
| FH m tarsus length | -0.192 | -0.665 – 0.259 | 0.235 | -0.816 | 0.415 |
| FH territory choice (near): PM m tarsus length | 0.163 | -0.442 – 0.775 | 0.310 | 0.528 | 0.598 |

Table S10. Parameter estimates of the generalized linear mixed models for the number of pied flycatchers [FH] nestlings in relation to phenotype of great tit [PM] (a) females [f] and (b) males [m] in year 2018.

a)

| **Fixed estimates** | **Estimate** | **95 % CI** | **SE** | **Z** | **p-value** |
| --- | --- | --- | --- | --- | --- |
| (Intercept) | -1.942 | -10.815 – 6.945 | 4.524 | -0.429 | 0.668 |
| FH territory choice (near) | 2.160 | -9.205 – 13.854 | 5.875 | 0.368 | 0.713 |
| PM f tarsus length | 0.087 | -0.292 – 0.481 | 0.197 | 0.442 | 0.658 |
| FH territory choice | 0.044 | -0.279 – 0.374 | 0.166 | 0.264 | 0.792 |
| FH m tarsus length | 0.047 | -0.296 – 0.388 | 0.174 | 0.270 | 0.787 |
| FH territory choice (near): PM f tarsus length | -0.096 | -0.624 – 0.417 | 0.265 | -0.362 | 0.717 |

b)

| **Fixed estimates** | **Estimate** | **95 % CI** | **SE** | **Z** | **p-value** |
| --- | --- | --- | --- | --- | --- |
| (Intercept) | 4.919 | -12.432 – 22.514 | 8.908 | 0.552 | 0.581 |
| FH territory choice (near) | -8.237 | -25.015 – 8.131 | 8.448 | -0.975 | 0.330 |
| PM m tarsus length | -0.207 | -0.764 – 0.346 | 0.283 | -0.731 | 0.465 |
| FH territory choice | -0.057 | -0.434 – 0.322 | 0.193 | -0.295 | 0.768 |
| FH m tarsus length | 0.132 | -0.197 – 0.467 | 0.169 | 0.781 | 0.435 |
| FH territory choice (near): PM m tarsus length | 0.364 | -0.354 – 1.099 | 0.371 | 0.982 | 0.326 |

Table S11. Parameter estimates of linear mixed model for pied flycatcher nestling tarsus length in relation to phenotype of great tit [PM] (a) females [f] and (b) males [m] in year 2017.

a)

| **Fixed effects** | **Estimate** | **95 % CI** | | **SE** | | **DF** | **t-value** | | | **p-value** | |  |  |
| --- | --- | --- | --- | --- | --- | --- | --- | --- | --- | --- | --- | --- | --- |
| (Intercept) | 5.359 | -4.508 – 15.226 | | 4.969 | | 94 | 1.078 | | | 0.284 | |  |  |
| FH territory choice (near) | 0.129 | -9.960 -10.218 | | 4.733 | | 15 | 0.028 | | | 0.979 | |  |  |
| PM f tarsus length | 0.123 | -0.183 – 0.430 | | 0.144 | | 15 | 0.859 | | | 0.404 | |  |  |
| FH brood size | 0.088 | -0.045 – 0.222 | | 0.062 | | 15 | 1.417 | | | 0.177 | |  |  |
| FH f tarsus length | 0.198 | -0.094 – 0.490 | | 0.137 | | 15 | 1.444 | | | 0.169 | |  |  |
| FH m tarsus length | 0.347 | -0.017 – 0.712 | | 0.171 | | 15 | 2.032 | | | 0.060 | |  |  |
| FH territory choice (near): PM f tarsus length | -0.005 | -0.454 – 0.444 | | 0.211 | | 15 | -0.023 | | | 0.982 | |  |  |
|  |  |  | |  |  | | | |  | |  | |  |
| **Random effects** | **SD** | **95 % CI** | **Residual** | | |  | |  | | |  | | |
| FH nest | 0.276 | 0.164 – 0.464 | 0.384 | | |  | |  | | |  | | |

b)

| **Fixed effects** | **Estimate** | **95 % CI** | | **SE** | | **DF** | **t-value** | | | **p-value** | |  |  |
| --- | --- | --- | --- | --- | --- | --- | --- | --- | --- | --- | --- | --- | --- |
| (Intercept) | 0.481 | -10.949 – 11.910 | | 5.752 | | 89 | 0.084 | | | 0.934 | |  |  |
| FH territory choice (near) | 8.186 | -3.688 – 20.060 | | 5.496 | | 13 | 1.489 | | | 0.160 | |  |  |
| PM m tarsus length | 0.211 | -0.169 – 0.591 | | 0.176 | | 13 | 1.201 | | | 0.251 | |  |  |
| FH brood size | 0.187 | 0.018 – 0.356 | | 0.078 | | 13 | 2.387 | | | 0.033 | |  |  |
| FH f tarsus length | 0.203 | -0.122 – 0.528 | | 0.150 | | 13 | 1.352 | | | 0.199 | |  |  |
| FH m tarsus length | 0.449 | 0.051 – 0.848 | | 0.185 | | 13 | 2.435 | | | 0.030 | |  |  |
| FH territory choice (near): PM m tarsus length | -0.348 | -0.860 – 0.165 | | 0.237 | | 13 | -1.464 | | | 0.167 | |  |  |
|  |  |  | |  |  | | | |  | |  | |  |
| **Random effects** | **SD** | **95 % CI** | **Residual** | | |  | |  | | |  | | |
| FH nest | 0.267 | 0.156 – 0.457 | 0.376 | | |  | |  | | |  | | |

Table S12. Parameter estimates of linear mixed model for pied flycatcher nestling tarsus length in relation to phenotype of great tit [PM] (a) females [f] and (b) males [m] in year 2018.

a)

| **Fixed effects** | **Estimate** | **95 % CI** | | **SE** | | **DF** | **t-value** | | | **p-value** | |  |  |
| --- | --- | --- | --- | --- | --- | --- | --- | --- | --- | --- | --- | --- | --- |
| (Intercept) | 3.532 | -2.245 – 9.309 | | 2.922 | | 139 | 1.209 | | | 0.229 | |  |  |
| FH territory choice (near) | 11.937 | 4.371 -19.503 | | 3.666 | | 24 | 3.256 | | | 0.003 | |  |  |
| PM f tarsus length | 0.394 | 0.144 – 0.649 | | 0.122 | | 24 | 3.241 | | | 0.004 | |  |  |
| FH brood size | -0.062 | -0.147 – 0.023 | | 0.041 | | 24 | -1.504 | | | 0.146 | |  |  |
| FH f tarsus length | 0.185 | -0.032 – 0.402 | | 0.105 | | 24 | 1.759 | | | 0.091 | |  |  |
| FH m tarsus length | 0.188 | -0.041 – 0.417 | | 0.111 | | 24 | 1.696 | | | 0.103 | |  |  |
| FH territory choice (near): PM f tarsus length | -0.542 | -0.884 – -0.200 | | 0.165 | | 24 | -3.275 | | | 0.003 | |  |  |
|  |  |  | |  |  | | | |  | |  | |  |
| **Random effects** | **SD** | **95 % CI** | **Residual** | | |  | |  | | |  | | |
| FH nest | 0.220 | 0.143 – 0.339 | 0.349 | | |  | |  | | |  | | |

b)

| **Fixed effects** | **Estimate** | **95 % CI** | | **SE** | | **DF** | **t-value** | | | **p-value** | |  |  |
| --- | --- | --- | --- | --- | --- | --- | --- | --- | --- | --- | --- | --- | --- |
| (Intercept) | 21.268 | 8.925 – 33.611 | | 6.237 | | 127 | 3.410 | | | 0.001 | |  |  |
| FH territory choice (near) | -9.489 | -22.567 – 3.588 | | 6.289 | | 21 | -1.509 | | | 0.146 | |  |  |
| PM m tarsus length | -0.498 | -0.926 – -0.070 | | 0.206 | | 21 | -2.419 | | | 0.025 | |  |  |
| FH brood size | -0.061 | -0.162 – 0.040 | | 0.049 | | 21 | -1.252 | | | 0.224 | |  |  |
| FH f tarsus length | 0.066 | -0.213 – 0.346 | | 0.134 | | 21 | 0.492 | | | 0.628 | |  |  |
| FH m tarsus length | 0.421 | 0.459 – 0.684 | | 0.126 | | 21 | 3.335 | | | 0.003 | |  |  |
| FH territory choice (near): PM m tarsus length | 0.420 | -0.154 – 0.994 | | 0.276 | | 21 | 1.521 | | | 0.143 | |  |  |
|  |  |  | |  |  | | | |  | |  | |  |
| **Random effects** | **SD** | **95 % CI** | **Residual** | | |  | |  | | |  | | |
| FH nest | 0.253 | 0.165 – 0.387 | 0.351 | | |  | |  | | |  | | |

1. b)


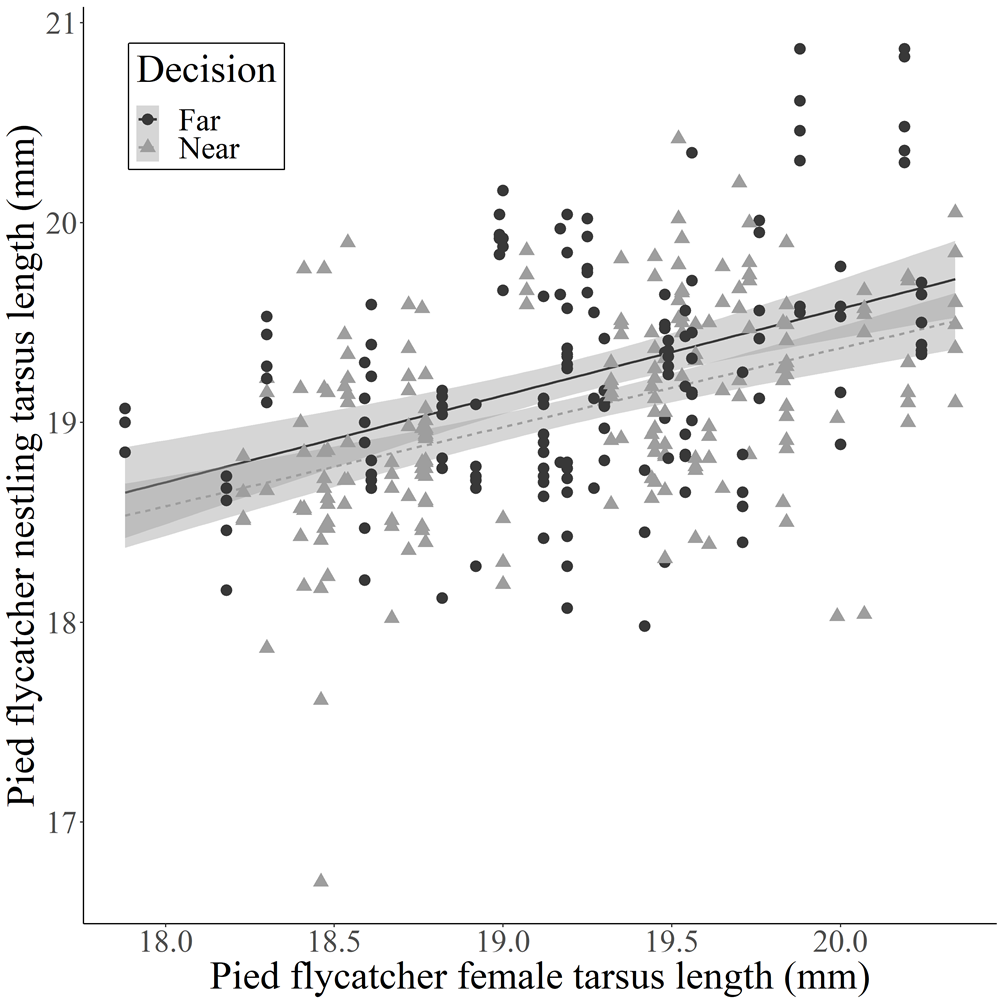

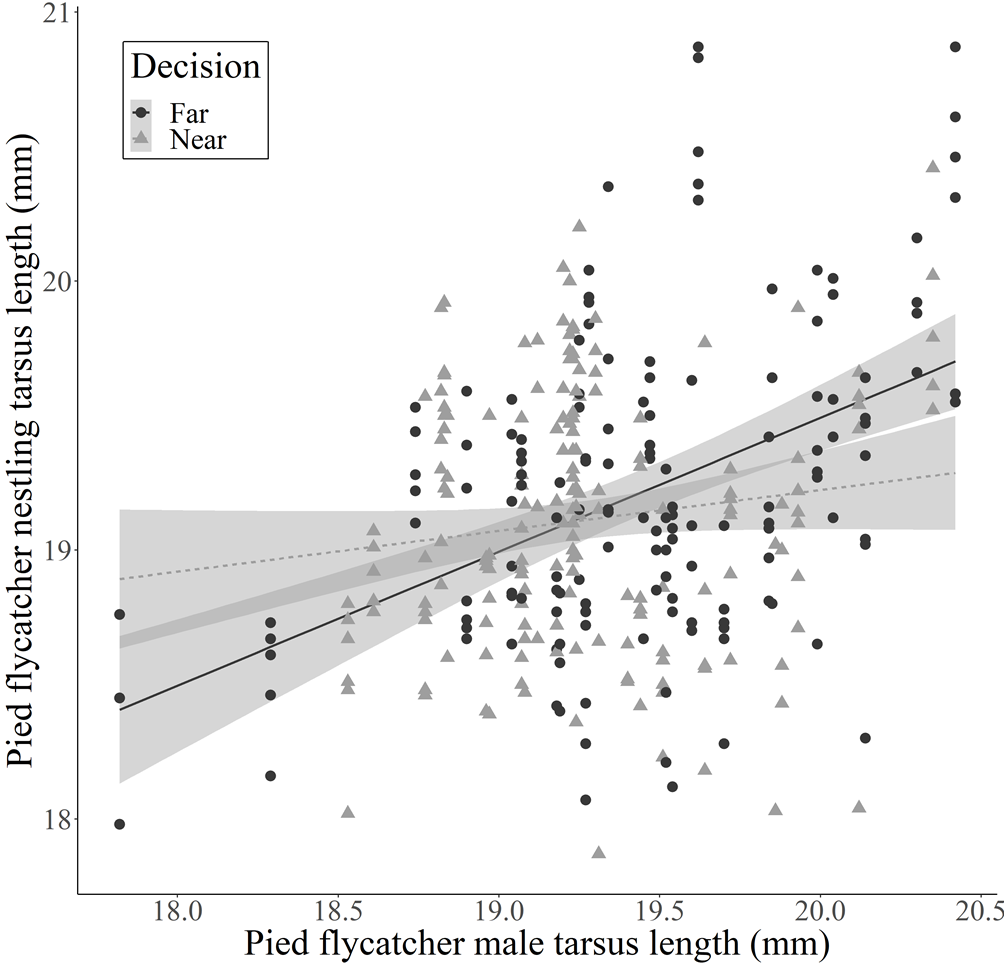


Figure S1 . Associations between **a)** piedflycatcher female **b)** pied flycatcher male tarsus length with pied flycatcher fledgling tarsus length far (black solid line) and near (grey dashed line) from great tit nest with 95% confidence interval.


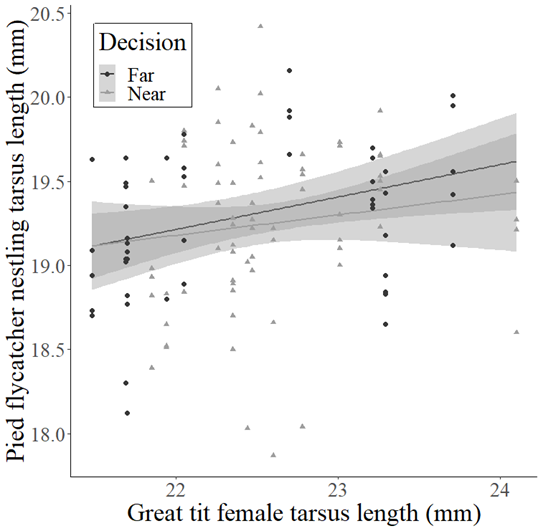


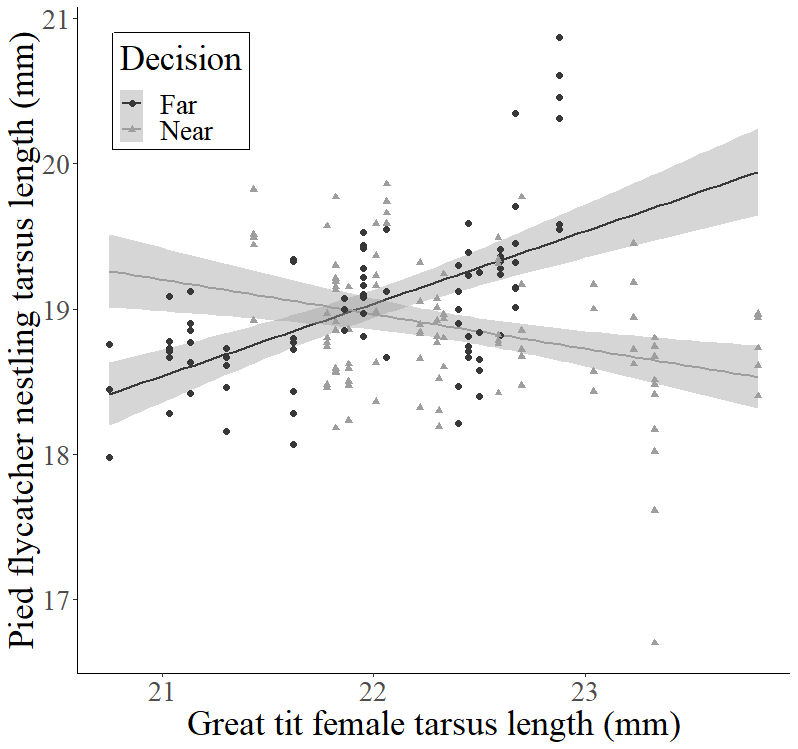


Figure S2. Association between great tit female tarsus length (mm) and pied flycatcher tarsus length (mm) near (gray dashed line) and far (black solid line) from great tit nest with 95% confidence intervals in years a. 2017 and b. 2018
